# A Comparative Study of Thermal Aging Effect on the Properties of Silicone-Based and Silicone-Free Thermal Gap Filler Materials

**DOI:** 10.3390/ma14133565

**Published:** 2021-06-25

**Authors:** A S M Raufur Chowdhury, Monjur Morshed Rabby, Mehzabeen Kabir, Partha Pratim Das, Rabin Bhandari, Rassel Raihan, Dereje Agonafer

**Affiliations:** 1 Department of Mechanical and Aerospace Engineering, University of Texas at Arlington, Arlington, TX 76019, USA; monjurmorshed.rabby@mavs.uta.edu (M.M.R.); mehzabeenbinte.kabir@mavs.uta.edu (M.K.); parthapratim.das@mavs.uta.edu (P.P.D.); rabin.bhandari@mavs.uta.edu (R.B.); mdrassel.raihan@uta.edu (R.R.); agonafer@uta.edu (D.A.); 2 Institute of Predictive Performance and Methodologies, University of Texas at Arlington Research Institute, Fort Worth, TX 76118, USA

**Keywords:** thermal gap filler material, dynamic mechanical analysis, thermomechanical analysis, differential scanning calorimetry, Fourier transform infrared spectroscopy, broadband dielectric spectroscopy

## Abstract

Thermal conductive gap filler materials are used as thermal interface materials (TIMs) in electronic devices due their numerous advantages, such as higher thermal conductivity, ease of use, and conformity. Silicone is a class of synthetic materials based on a polymeric siloxane backbone which is widely used in thermal gap filler materials. In electronic packages, silicone-based thermal gap filler materials are widely used in industries, whereas silicone-free thermal gap filler materials are emerging as new alternatives for numerous electronics applications. Certainly, characterization of these TIMs is of immense importance since it plays a critical role in heat dissipation and long-term reliability of the electronic packages. Insubstantial studies on the effects of various chemical compounds on the properties of silicone-based and silicone-free TIMs has led to this study, which focuses on the effect of thermal aging on the mechanical, thermal, and dielectric properties of silicone-based and silicone-free TIMs and the chemical compounds that cause the changes in properties of these materials. Characterization techniques such as dynamic mechanical analysis (DMA), thermomechanical analysis (TMA), differential scanning calorimetry (DSC), Fourier transform infrared spectroscopy (FTIR), and broadband dielectric spectroscopy (BbDS) are used to study the mechanical, thermal, and dielectric characteristics of these TIMs, which will guide towards a better understanding of the applicability and reliability of these TIMs. The experiments demonstrate that upon thermal aging at 125 °C, the silicone-free TIM becomes hard, while silicone-based TIM remains viscoelastic, which indicates its wide applicability to higher temperature applications for a long time. Though silicone-based TIM displays better mechanical and thermal properties at elevated temperatures, dielectric properties indicate low conductivity for silicone-free TIM, which makes it a better candidate for silicone-sensitive applications where higher electric insulation is desired.

## 1. Introduction

**Figure 1:**
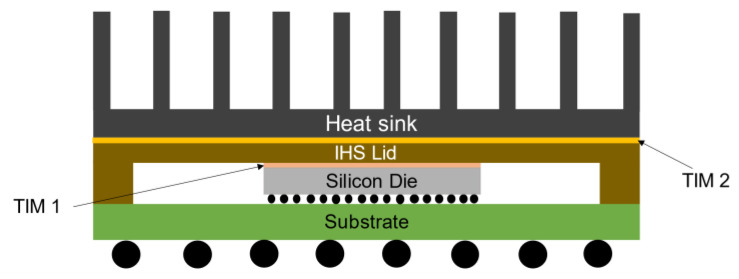
Schematic of electronic package with heat sink and IHS.

Thermal interface material plays a significant role in the electronic packages to enhance the heat transfer between contact surfaces. Due to miniaturization, heat dissipation from the electronic devices has become one of the limiting factors in device performance and reliability. For the optimum performance and better reliability of a device, it is important to dissipate heat efficiently from the device during its normal operation. When two mating parts are attached in an electronic device, voids can be formed in between these two mating surfaces. Generally, these voids are filled with air, which increases thermal contact resistance. Heat dissipating devices cannot dissipate heat efficiently without having good thermal contact between the mating parts. Thermal interface materials (TIMs) are essential for good contact between the adjacent surfaces and minimizing the thermal contact resistance. Thermal gap fillers are used to fill voids between mating parts such as silicon die and heat sinks, and it is important to study the thermal properties of this material. The elevated temperature in the electronic devices has an impact on the properties, reliability, and the thermal performance of the thermal interface materials (TIMs). From a lifecycle viewpoint, the long-term reliability of the thermal interface materials (TIMs) is a concern for the industry [1]. The package in which a TIM is mounted, the environment it is subjected to, and the nature of its service all influence the long-term reliability of TIMs. As a result, in two entirely different devices, the same TIM can be applied, and in one of the devices it will fail prematurely while in the other it will increase efficiency [2]. Different types of thermal interface materials (TIMs) are used in the industry, such as thermal greases, phase change materials (PCM), elastomer pads, thermal gap filler materials, thermal pads, and epoxies [3]. An electronic package with integrated heat spreader (IHS) and heat sink is shown in Figure 1. The schematic demonstrates that TIM 1 is placed in between silicon die and IHS, and TIM 2 is placed in between IHS and heat sink to minimize thermal resistance. In general, polymer-based TIMs and solder-based TIMs are used for the TIM 1 interface.

Polymer-based TIMs are primarily comprised of elastomeric resin and thermally conductive fillers [4]. Elastomers are special polymers that have both high viscosity and elasticity. Depending on the effectiveness and application, a specific TIM is chosen for a device. TIMs used between high-power devices and heat sink need to be electrically insulating, and at the same time, these TIMs need to have high thermal conductivity to provide efficient thermal performance [3]. For electronic devices, thermally conductive and electrically insulating polymer matrix composites are used to acquire optimum performance and reliability of electronic devices. There are different fillers which possess the aforementioned properties, such as aluminum nitride, silicon carbide, and alumina [5]. The dielectric constant of materials has a significant impact on device performance. Materials with low dielectric constant reduce the signal delay and enhance device performance [3].

Silicone-based TIMs are broadly used in electronic devices. Silicone is a class of synthetic material based on a polymeric siloxane backbone containing silicon, and oxygen atoms attached to the silicon atoms. It is categorized as elastomers, fluids, or resins depending on the range of crosslinking. Silicone resins are highly crosslinked structures, and at room temperature they can be either liquid or solid. Silicone provides chemical and thermal stability, low glass transition temperature, and low viscosity and near-zero surface shear viscosity [6]. Silicone-free thermal interface materials are emerging as a new choice of thermal interface materials for industries for the silicone-sensitive devices in many applications, such as optical, medical, and sensor devices, preventing manufacturers from using silicone-based materials [7].

Characterization of the properties of thermal interface materials is primarily important since it plays a vital role in the thermal dissipation and the lifecycle of the of the electronic packages. Goel et al. identified that there is a need for developing new techniques of mobile device application since existing methods such an ASTM D5470 testing do not include the operating conditions for mobile devices. They analyzed the end-of-life performance of thermal grease and phase change materials [8]. Fabris et al. found that performance of the commercial TIM degrades with the addition of large quantities of carbon nanotubes (CNTs), while on the other hand, at higher pressure, CNT and silicone oil mixtures showed improved performance [9]. Thermal conductive gap filler materials should possess good thermal conductivity, high-temperature stability, and elasticity at low temperatures. However, mechanical properties, for example dynamic modulus, creep, and stress relaxation, have an impact on the long-time reliability of the thermal interface materials (TIMs). The characterization of the beginning of life performance of TIMs is not sufficient since performance can degrade with usage [8]. Roy et al. used isothermal aging and thermal cycling to determine the lifecycle and performance degradation of low melting alloys (LMA). They discovered that low melting alloys (LMA) can withstand 2700 h of thermal aging at 130 °C and 1400 cycles between −40 and 80 °C without considerable performance degradation [10]. Misrak et al. studied the mechanical properties of commercially available, thermally conductive gap filler materials. They performed a high-temperature storage test at 125 °C for 720 h to determine the change in mechanical properties of the TIMs [11]. Isothermal ageing or bake tests, also referred to as high-temperature thermal storage tests, are designed to simulate high temperatures experienced by the TIMs [12]. Due et al. mentioned that there is lack of understanding about the mechanism that causes TIM failure as there are not enough studies on the basic physics of TIM degradation [2]. Additionally, moisture present in the air can affect the modulus, strength, and damping properties of polymers [13]. Due to the scarcity in understanding the behavior of TIMs in the packages under different circumstances, the impact of chemical compounds on the properties of the TIMs is still an ongoing research effort.

This study focuses on the effect of thermal aging on silicone-based and silicone-free thermal gap filler materials and the effect of different chemical compounds on the silicone-based and silicone-free thermal gap filler materials. In this study, isothermal aging is performed at 125 °C for 2000 h for both the thermal gap filler materials to investigate the thermal aging effect on the gap filler materials. Properties such as storage modulus, loss modulus, complex modulus, thermal expansion, and dielectric properties of thermal gap filler materials are analyzed, and the causes of the change in the properties are investigated to better understand how TIMs’ characteristics change with thermal aging. Established characterization techniques, e.g., dynamic mechanical analysis (DMA), thermomechanical analysis (TMA), differential scanning calorimetry (DSC), Fourier transform infrared spectroscopy (FTIR), and broadband dielectric spectroscopy (BbDS), are used in this study to analyze the material state of the TIMs. Finally, it can be concluded that the core purpose of this analysis relies on the change of mechanical, thermal, and dielectric properties of silicone-based and silicone-free TIMs after thermal aging. Additionally, the physics behind those changes are explained by different characterization techniques applied in this study.

## 2. Materials and Methods

### 2.1. Materials

**Figure 2:**
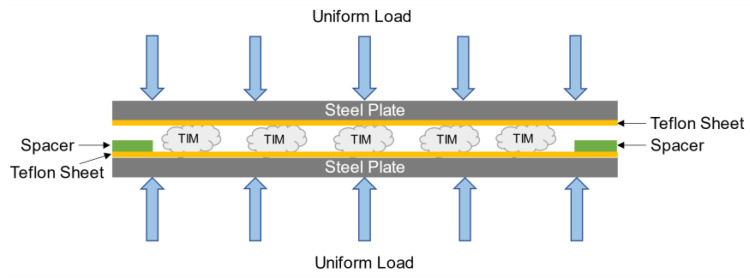
Schematic of TIM sample preparation assembly.

This research characterizes commercially available silicone-based and silicone-free thermal gap filler materials. Throughout this article, silicone-based and silicone-free TIMs are referred to as TIM A and TIM B, respectively. Steel plates, Teflon boards, double sided adhesive tape, spacers, and clamps were used to prepare TIM samples. Teflon sheets were adhered to one side of two steel plates with double sided adhesive tape. TIM samples were dispensed on one of the plates’ Teflon board, and spacers were mounted on both sides of the plates. The other steel plate was affixed to the end. Teflon was used to prevent gap filler materials from adhering to the steel plates. Pressure was applied using clamps on both sides of the plates and the TIMs were cured for 24 h at room temperature (~25 °C). TIM B samples were prepared using the same procedure. The schematic in Figure 2 shows TIM sample preparation assembly to obtain the required dimension of the sample. Isothermal aging at 125 °C (high-temperature storage life, JESD22-A103E, Condition A) was performed for 2000 h for TIM A and TIM B to analyze changes in properties of the TIMs. An EC 127 Temperature Test Chamber was used to perform the thermal aging of TIMs. In this study, for both the TIMs, the samples that are not thermal-aged are referred to as preaged, and after thermal aging, the samples are referred to as thermal-aged.

### 2.2. Methods

#### 2.2.1. Dynamic Mechanical Analysis (DMA)

**Figure 3:**
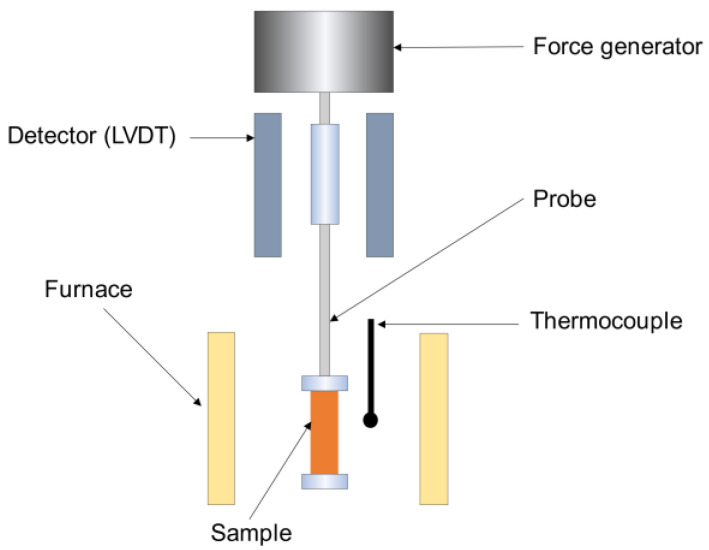
Schematic of dynamic mechanical analyzer (tension mode) [16].

Mechanical properties of a polymer can be measured using the dynamic mechanical analysis (DMA) technique applying variable stress or strain applied at range over time. It is a technique which provides a relationship between temperature and the mechanical properties of a sample related to vibration load (or strain) of a material [14]. It consists of a force generator which applies a sinusoidal force on the sample though the probe and its deformation is detected by a LVDT detector. The schematic of the dynamic mechanical analyzer is shown in Figure 3. The relation between applied stress and measured strain is considered to calculate viscoelastic properties of a material. Since most polymers are viscoelastic materials, they have both elastic and viscous components. DMA measures the viscoelastic characteristics, such as storage modulus (*E*′), loss modulus (*E*″), loss tangent (*Tanδ*), and their dependency on frequency and temperature. Storage modulus is defined as the stored energy and loss modulus is defined as energy lost in the form of heat. The relations between complex modulus, storage modulus, and loss modulus are shown in Equations (1) and (2) [11,15]. Equation (3) [11,15] shows the relation of loss tangent with storage and loss modulus.

(1) $$E^{*}=E^{\prime }+iE^{\prime \prime }$$

(2) $$\left|E^{*}\right|=\sqrt{{E^{\prime }}^{2}+{E^{\prime \prime }}^{2}}$$

(3) $$Tan\delta =\frac{E^{\prime \prime }}{E^{\prime }}$$

For dynamic mechanical analysis of the thermal gap filler materials, DMA 7100 was used. Several modes such as tension, compression, shear, and dual cantilever bending modes are available in DMA 7100, and depending on the sample shape and desired modulus, a suitable mode needs to be selected. By using different attachments, *E*″, *E*′, and *Tanδ* can be measured for different materials. The storage and loss modulus are measured for silicone-based and silicone-free thermal gap filler materials using the dynamic mechanical analyzer. In this analysis, tensile attachment was used. For each TIM, at least four tests are performed for dynamic mechanical analysis. To perform the analysis, both preaged and thermal-aged TIM A and TIM B samples’ lengths were ~30 mm, and widths were 6 to 9 mm. The thickness of preaged TIM A samples was ~2.7 mm, and the thickness of thermal-aged TIM A and TIM B samples was ~1 mm in this study. When the geometry factor is very large, the sample cannot be extended, resulting in a small measurement amount. On the other hand, if the geometry factor is too small, the sample deformation amount is large even when a small force amplitude is applied, resulting in the set strain amplitude being exceeded [14]. TIM A was the softer material of the two TIMs, and for this material, thicker samples were prepared to have the geometric factor with the range of the DMA. The properties were measured from 20 to 170 °C, at a 2 °C/min heating rate, for 0.5, 1, 2, 5, and 10 Hz frequencies. For this study, minimum force of 50 mN and force gain of 1.5 were used, and properties of the TIMs were analyzed at 1 Hz dynamic frequency [4].

#### 2.2.2. Thermomechanical Analysis (TMA)

**Figure 4:**
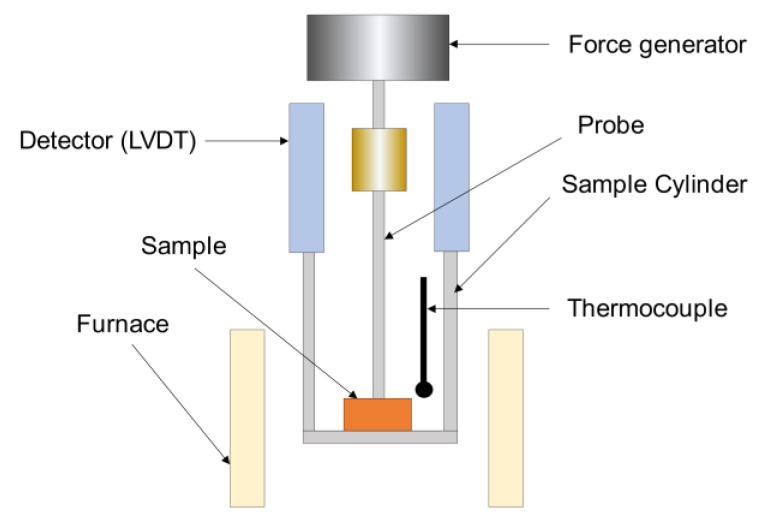
Schematic of the thermomechanical analyzer (TMA) [17].

In thermomechanical analysis, deformation of a sample is measured under non-oscillating stress with respect to temperature or time [17]. The thermomechanical analyzer was used to measure the coefficient of thermal expansion (CTE) and glass transition temperature (T_g_) of the materials. In this technique, materials are characterized by measuring the deformation of material with respect to temperature. The schematic of the thermomechanical analyzer is shown in Figure 4. It consists of a furnace to heat or cool the sample and the force is applied on the sample by a force generator through the probe. The deformation of the sample is measured by the LVDT detector, and the results are used to compute CTE and T_g_ of the samples. A compression probe or a tensile attachment is used for the analysis. TMA is also capable of measuring modulus for thin and soft samples, however, in this analysis, it is used to measure the thermal expansion behavior of the thermal gap filler materials.

The thermomechanical analyzer TMA/SS6000 was used to perform the thermomechanical analysis of the thermal gap filler materials. Thermal expansion with the change of temperature was measured for both the TIMs using the thermomechanical analyzer. TIM A and TIM B specimens, with dimensions of height and width of ~7 mm and thickness of ~1 mm, were placed on top of the quartz stage. For this analysis, a compression probe was used for measuring the thermal expansion of the thermal gap filler materials. To ensure contact between the sample and the probe throughout the analysis, a load of 10 mN was applied on the sample. The measurements of TIMs were performed from 25 to 130 °C at a heating rate of 3 °C/min to understand the thermal expansion behavior. Isothermal hold was performed to bring the sample to the desired temperature. Each measurement was performed at least three times. The tip of the probe and the quartz stage were cleaned with ethanol after each experiment to remove the residual.

#### 2.2.3. Differential Scanning Calorimetry (DSC)

Observing thermal transitions is a major aspect of characterizing electronic materials to study thermal stability. Differential scanning calorimetry measures the difference in the quantity of heat required for increasing the temperature of the sample and reference with respect to temperature. It can also analyze the amount of heat absorbed or released during these transitions. The thermal properties of the polymer and the decomposition behavior were studied using differential scanning calorimetry. It also detects the transitions of melts, glass transitions, phase changes, and curing [18]. In a heat flux DSC system, the sample and reference are heated at an equal rate from a single heat source. Temperature difference (*dt*) between the pans was recorded and converted to a power difference (ΔP). This power difference provides the difference in heat flow (dH) [19].

(4) ΔP=dH/dt

Using DSC, the heat flow in a sample is measured, which is attributed to how the material absorbs or generates energy at the molecular level when heat is applied. During heating, molecules inside the material start vibrating, however, they also rotate and translate, then the heat flow becomes higher. At the glass transition region, the material starts shifting its state from rigid to more flexible, which in turn increases the heat flow [20]. Thus, heat flow indicates changes in structure. Equation (5) is used to calculate the heat flow, where heat flow is *dH*/*dt*, heat capacity is *C_p_*, heating rate is *β*, and the mass of the sample is *M* [21,22,23].

(5) $$\frac{dH}{dt}=C_{p}\times \beta \times M$$

Discovery DSC 25 was used to analyze the thermal properties of the gap filler materials. A Tzero aluminum pan and a ramp from 40 °C temperature to 450 °C at a heating rate of 5 °C/min were used to investigate changes of thermal properties that occurred in this temperature range.

#### 2.2.4. Fourier Transform Infrared Spectroscopy (FTIR)

FTIR is a prominent technique to measure infrared emission and absorption spectra of materials. One of the most important advantages of the FTIR technique is that virtually all the compounds display their absorption/emissions in the IR spectral region, which can be quantitatively and qualitatively analyzed based on their properties [24]. Organic and inorganic materials can be analyzed using FTIR spectroscopy [11]. A Thermo Nicolet 6700 FTIR Spectrometer was used for evaluating the molecular properties in the thermal gap filler materials. For every sample, 32 scans were collected. The spectra were collected for a wavelength of 4000 to 650 cm^−1^. Using this spectrometer, spectra can be collected at multiple IR spectral ranges.

#### 2.2.5. Broadband Dielectric Spectroscopy (BbDS)

Broadband dielectric spectroscopy is the analysis of interaction of the electromagnetic waves with matter. It has become a very prominent method to determine the dielectric properties of materials. Using this technique, dielectric properties of a material can be measured with reference to frequency and temperature. This technique provides information about molecular dynamics, dielectric constant, and dielectric loss of different polymer materials [25]. Morphological heterogeneity, the interactions of structural and electrical properties between the particles, and the shape as well as the orientation of the combining phases are several different factors that can change the dielectric spectra of a heterogeneous material.

**Figure 5:**
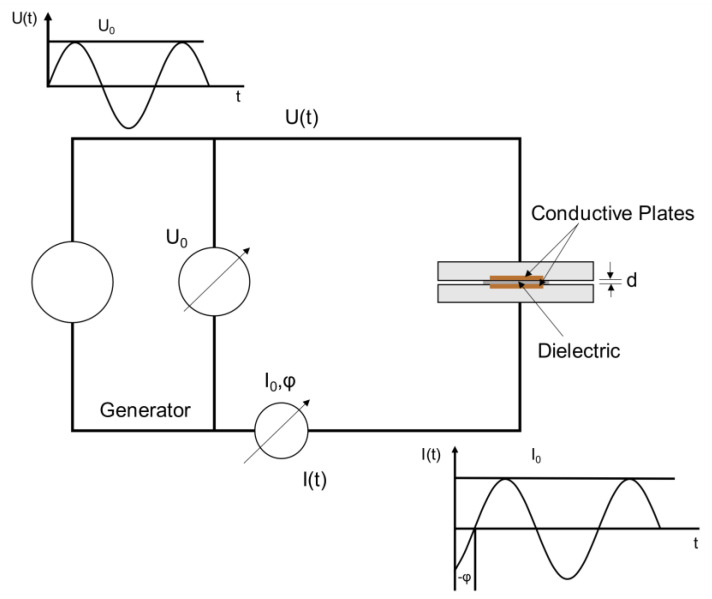
Principle of dielectric measurement [26].

**Figure 6:**
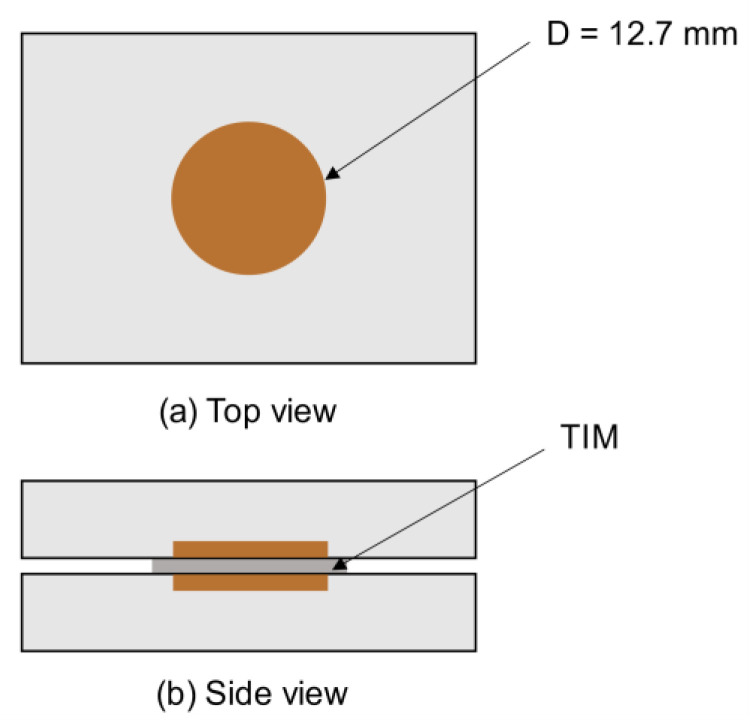
(**a**) Conductive plates on the electrode, (**b**) TIM placed in between the electrodes.

To perform dielectric studies, a parallel plate arrangement was created by placing the sample between the electrode blocks connected to the analyzer of the NOVOCONTROL^TM^ unit. Uniform contact of electrodes with the sample was ensured by clamping the blocks and the impedance, and capacitance was measured as function of frequency with high precision. A schematic of dielectric properties’ measurement setup is shown in Figure 5. The conductive plates in the electrode and the TIM placed in between the conductive plates are shown in Figure 6. In this study, a 1-volt AC current was applied and a frequency of 10^−1^ to 10^6^ Hz was used for dielectric response analysis. The measured permittivity of the sample is given by:

(6) $$\varepsilon_{r}{}^{*}\left(\omega \right)=\varepsilon_{r}^{\prime }+j{\varepsilon_{r}}^{\prime \prime }=\frac{C^{*}}{C_{0}}$$

(7) $$C_{0}=\varepsilon_{0}\frac{A}{d}$$

where ε_r_* is complex permittivity, and *ε_r_*′ and *ε_r_*″ represent real and imaginary parts of measured ε_r_*. The permittivity of the free space is presented by $\varepsilon_{0}\left(F\;m^{-1}\right)$ and the capacitance of the free space is presented by *C*_0_(*F*) [26].

## 3. Results and Discussion

### 3.1. Dynamic Modulus

In general, gap filler materials are viscoelastic in nature and have rubber-like properties. Dynamic mechanical analysis was used to confirm their viscoelastic nature. At glass transition temperature (T_g_), polymeric material shows state changes. Decreasing the temperature of a polymer below T_g_ leads the material to a brittle state, like a glass, whereas above the T_g_, the material state is more like a rubber [27]. Therefore, elastomer should behave like rubber at room temperature as the glass transition temperature is very low for elastomer [28]. For this reason, compared to the other polymers, elastomers exhibit a decreased modulus of elasticity and higher strain at break. In this study, dynamic mechanical analysis (DMA) was performed for preaged and thermal-aged TIM A and TIM B samples. Measurements of 1 Hz were used to compare the properties of the TIM A and TIM B samples. TIM A preaged samples were challenging to mount on the DMA tensile attachment fixture due to the softness of the sample, while on the other hand, TIM A thermal-aged samples were easier to mount on the DMA tensile fixture as they are not as soft as the preaged samples. Stiffness of TIM B preaged and thermal-aged samples was higher than TIM A thermal-aged samples, and both TIM B preaged and thermal-aged samples were easier to mount on the fixture. Tests were performed in the range of 25 to 170 °C.

**Figure 7:**
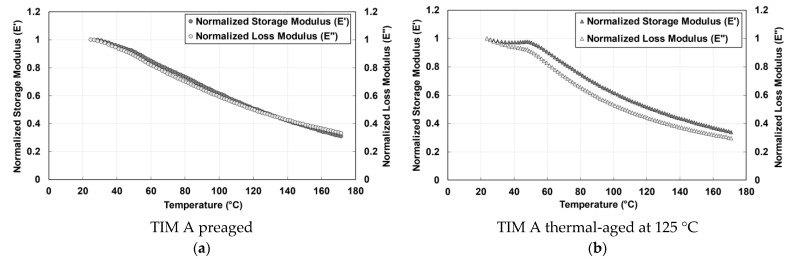
Storage modulus (*E*′) and loss modulus (*E*″), (**a**) TIM A preaged, and (**b**) (*E*″) TIM A thermal-aged at 125 °C.

The normalized storage and loss modulus of TIM A preaged and thermal-aged materials is shown in Figure 7. Storage modulus is used to define the elastic properties of a polymeric material. When the temperature increased, both TIM A and TIM B were found to lose their ability to store energy. When the energy storage capacity of the materials decreases with increasing temperature, the intermolecular bond of the materials weakens, and the stiffness of the materials decreases. It has been observed that the complex modulus of silicone-based thermal gap filler material normally decreases with heating. As previously stated, polymer chain scission occurred during heating for TIM A. Since during thermal aging the samples were held at 125 °C for 2000 h, the polymer chain has enough time to move and the polymer chain motion continues, resulting in entanglement among some polymer chains [29]. As a result, thermal-aged TIM A has a higher complex modulus than preaged TIM A. Loss modulus describes the viscous properties of a polymeric-based material and it represents how much energy is dissipating from the material [30]. The loss modulus of TIM A preaged and thermal-aged materials (Figure 7) decreases as temperature increases, because less force is required for deformation. 

**Figure 8:**
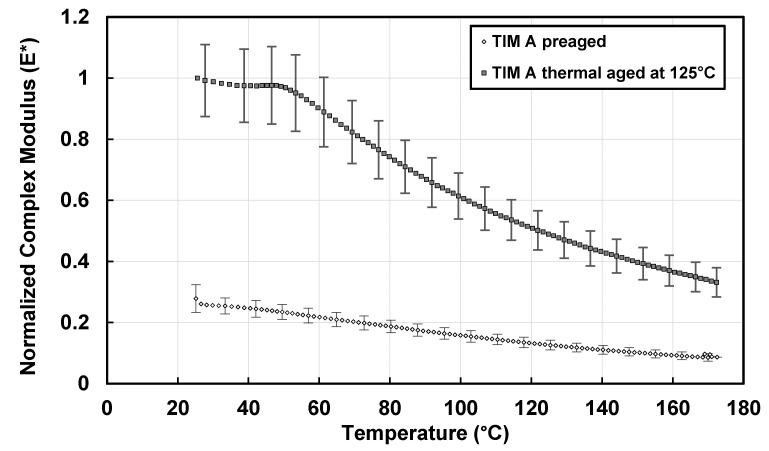
Complex modulus of TIM A.

Dynamic modulus, also referred to as complex modulus, is calculated from storage and loss modulus using Equation (2). The change in complex modulus with temperature of preaged and thermal-aged TIM A samples with the change of temperature is shown in Figure 8. The complex modulus of TIM A preaged and thermal-aged results are normalized against the value of the thermal-aged TIM A at 25 °C. The complex modulus of TIM A is increased when it is thermal-aged at 125 °C. From 50 to 170 °C, there is about a 65.3% decrease in complex modulus for the TIM A preaged sample, and there is about a 58.9% decrease in complex modulus for the TIM A thermal-aged samples.

**Figure 9:**
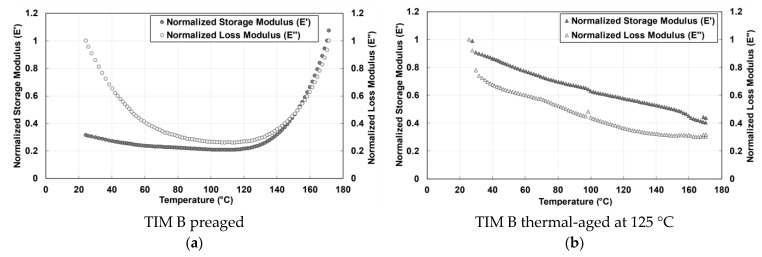
Storage modulus (*E*′) and loss modulus (*E*″), (**a**) TIM B preaged, and (**b**) TIM B thermal-aged at 125 °C.

The normalized storage and loss modulus of TIM B preaged and thermal-aged materials are shown in Figure 9. In Figure 9a, for TIM B preaged samples, the value of storage modulus and loss modulus decreases with the increase of temperature, however, after 115 °C temperature, the storage modulus and loss modulus increase as the material becomes stiffer. The reason behind the increase in stiffness of preaged TIM B is explained in the thermal properties section (Section 3.3).

**Figure 10:**
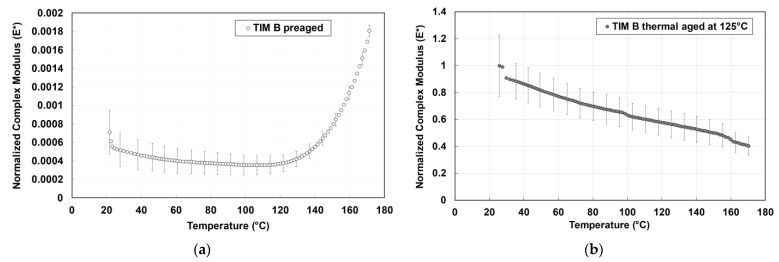
Complex modulus, (**a**) TIM B preaged, and (**b**) TIM B thermal-aged at 125 °C.

The complex modulus of TIM B preaged results and TIM B thermal-aged results is normalized against the thermal-aged TIM B value at 25 °C. Figure 10 shows the complex modulus of TIM B before aging and TIM B after thermal aging, respectively. The complex modulus of preaged TIM B is lower than thermal-aged TIM B. For preaged TIM B, the complex modulus decreased until 115 °C, and there was about a 16% decrease in complex modulus from 50 to 115 °C, while after 115 °C, the modulus started to increase rapidly with the increase of temperature. From Figure 10, it can be seen that the complex modulus of TIM B was much higher when it was thermal-aged at 125 °C. After thermal aging, the TIM B sample became hard, and the Vickers hardness of the sample was 59.6 HV, measured using a LECO LM300AT Microhardness Tester.

### 3.2. Thermal Expansion

**Figure 11:**
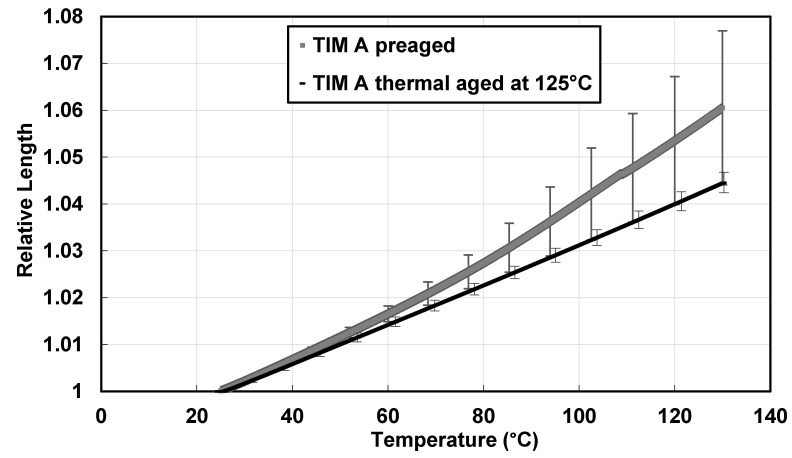
Change in relative length with temperature of TIM A.

To understand the thermal expansion behavior of the TIMs, the change in relative length of the sample was calculated with the change of temperature using the thermomechanical analyzer (TMA) from 25 to 130 °C. The change in relative length with temperature for preaged TIM A samples has been found to be greater than the ones thermal-aged at 125° (Figure 11). Preaged TIM A has a total thermal expansion of 4.84% from 50 to 130 °C, whereas thermal-aged TIM A has a total thermal expansion of 3.42%.

**Figure 12:**
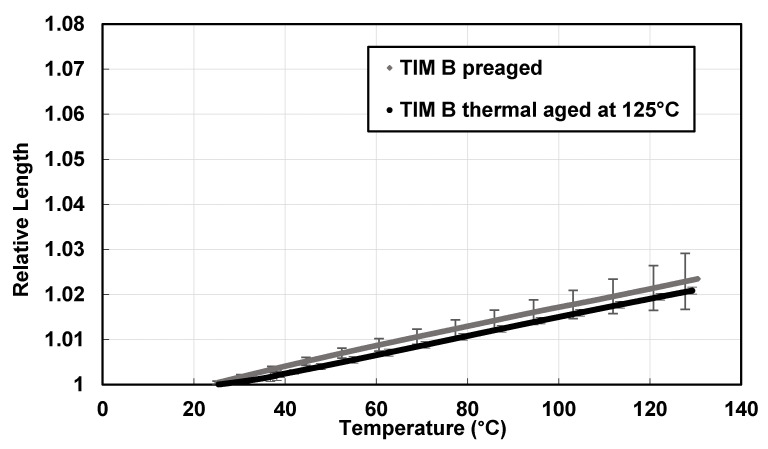
Change in relative length with temperature of TIM B.

The change in relative length with the change of temperature for TIM B is shown in Figure 12. In the case of TIM B, the changes in relative length with temperature for preaged and thermal-aged (at 125 °C) samples have been observed to be almost similar. Besides, thermal expansion from 50 to 130 °C for preaged TIM B was about 1.68% and for thermal-aged TIM B, it was about 1.62%.

From Figure 11 and Figure 12, it can be concluded that the thermal expansion of TIM A is significantly higher than TIM B for the preaged samples. For both the TIMs, thermal expansion was higher compared to that of copper, aluminum, or other underfill materials [4]. Thermal expansion of preaged TIM A was higher than thermal-aged TIM A, which can be correlated to dynamic mechanical analysis (DMA) results. Similarly, thermal expansion of TIM B decreased after thermal aging, which can also be correlated to the dynamic mechanical analysis (DMA) results.

### 3.3. Thermal Properties

In this study, thermal stability of two types of gap filler material, TIM A and TIM B, have been analyzed before and after thermal aging. Thermal interface materials are mostly elastomeric material and show significant physical property changes when exposed to heat. These changes in properties can have significant impacts on characteristics and lifecycle of the elastomeric materials [31,32,33]. Gap filler material (elastomeric material) has a glass transition temperature far below room temperature, and can be thought of as a single large molecule of macroscopic scale [34]. When the temperature drops, elastomers become harder and less flexible, and they lose their rubber-like properties entirely when the temperature reaches the glass transition temperature. On the other hand, at high temperatures near or above the service temperature limit, some irreversible chemical changes occur in elastomers. At this temperature, the polymer backbone may be subject to either chain scission, causing the elastomeric part to become softer, or the polymer molecules may undergo a crosslinking process, resulting in the material being more rigid, which in turn alters their thermal stability [34,35].

**Figure 13:**
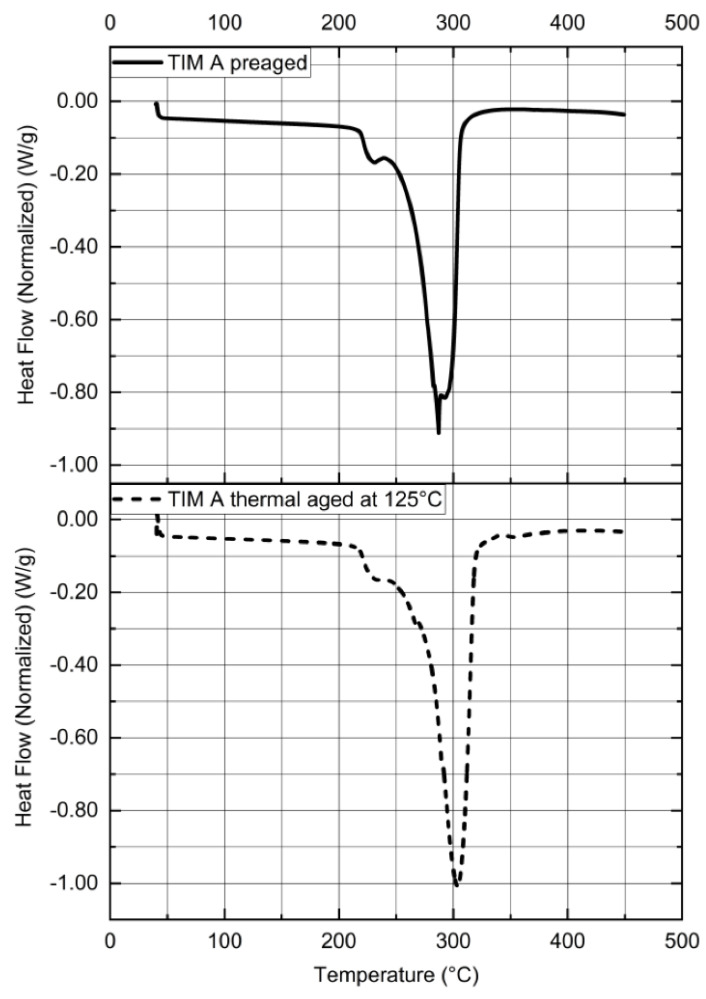
Normalized heat flow vs. temperature of TIM A.

**Figure 14:**
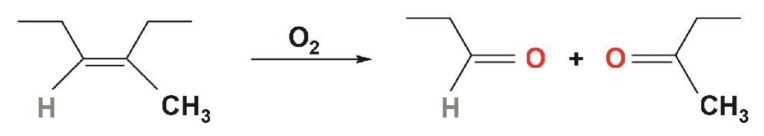
A representation of oxidative softening-chain scission [33].

From the DSC thermograph (Figure 13), it has been found that there is a sudden drop in heat flow for TIM A at 225 °C, which indicates oxidative degradation with subsequent polymer chain scission. During thermal aging of the elastomeric material, depending on the microstructure of the elastomer, degradation will cause either hardening or softening. For example, polybutadiene generally undergoes oxidative hardening, while on the other hand, polyisoprene softens as it is exposed to heat and oxygen [29]. According to the literature, pendent bulky side groups in polymer chains create steric hinderance, which causes a radical recombination reaction, which is difficult to occur. Therefore, these polymers undergo strain softening because of the chain scission [31,32,33,36]. A representation of oxidative softening—chain scission is shown in Figure 14. For this reason, preaged TIM A shows softening during heating. Similar degradation has been observed in a DSC thermograph for thermal-aged TIM A.

**Figure 15:**
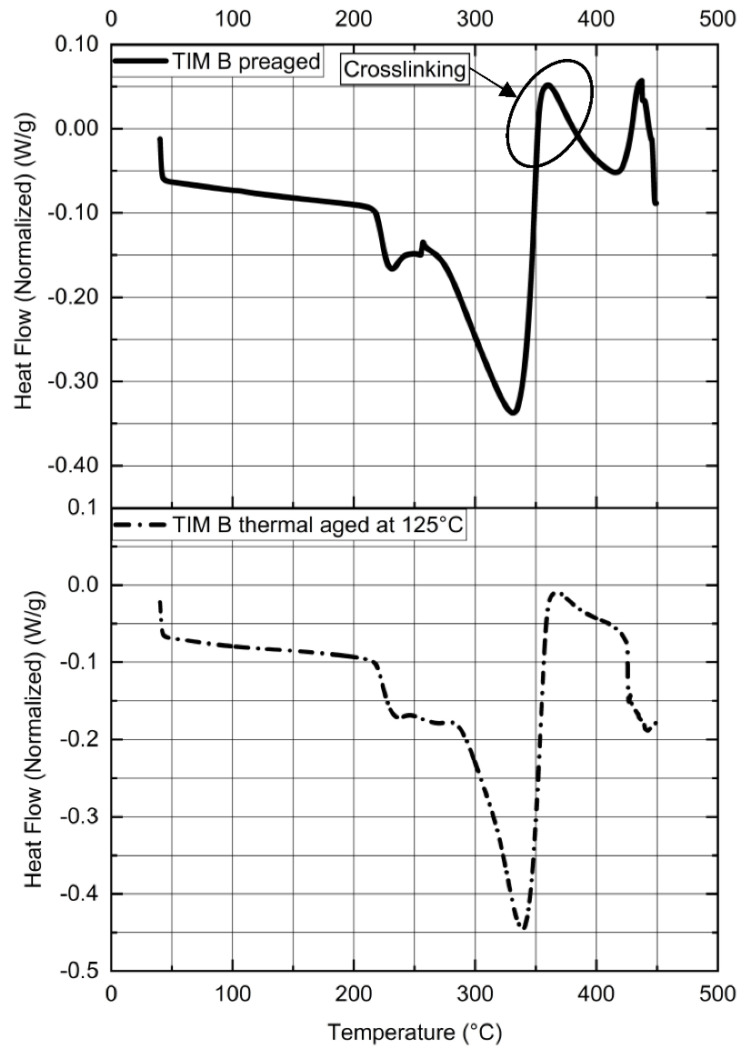
Normalized heat flow vs. temperature of TIM B.

However, TIM B material showed hardening when this material was exposed to heat. At 220 °C (Figure 15), TIM B showed similar transition because of the thermal degradation. However, an exothermic peak (because of reaction heat) has been observed in the DSC thermograph above 360 °C, which indicates the occurrence of a crosslinking reaction. The crosslinking will cause the elastomeric part to become hard. The crosslinking reaction occurs due to free radicals produced by heat. For this reason, after thermal aging, TIM B showed brittle-like behavior.

When the DSC experiment was performed for thermal-aged TIM B material with the same conditions, it was found that thermal ageing does not change the degradation temperature, however, a reduction in exothermic peak area (reaction heat) has been observed because most of the polymer chains were already crosslinked and there was less energy left for further reactions.

### 3.4. FTIR Spectra

**Figure 16:**
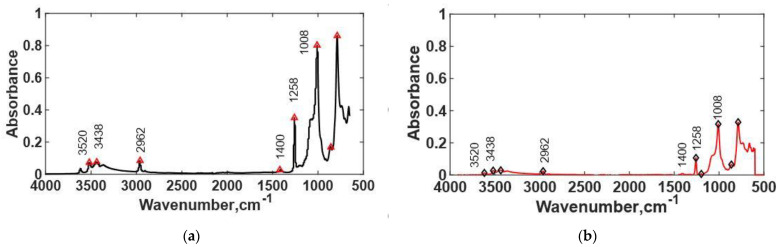
(**a**) FTIR spectra of TIM A preaged, and (**b**) FTIR spectra of TIM A thermal-aged at 125 °C.

FTIR spectra of TIM A preaged and TIM A thermal-aged at 125 °C are shown in Figure 16. TIM A preaged material showed an intense peak at a 1258 cm^−1^ wavelength, which indicates the presence of silicon organic compounds with a methyl group. Around 1260 cm^−1^, methyl groups attached to silicon atoms produced a distinctive deformation absorption [37]. The asymmetrical deformation was weaker and occurred around 1400 cm^−1^. As the material was thermal-aged, a certain decrease in the intensity at this wavelength was found, which indicates that polymer chain scissions occurred during thermal aging. The peak at the 2962 cm^−1^ wavelength was analyzed, and it shows that the CH_3_ and CH_2_ bonds decrease with thermal aging, which is a clear indication of polymer chain scission. The TIM A preaged sample also showed peaks at 3520 and 3438 cm^−1^ wavelengths, which define −OH bond (carboxylic acid) and −OH stretching vibration. In the thermal-aged sample, the intensity of the −OH bond decreased, which indicates that the −OH bond was broken at elevated temperature and free oxygen caused thermal oxidation.

**Figure 17:**
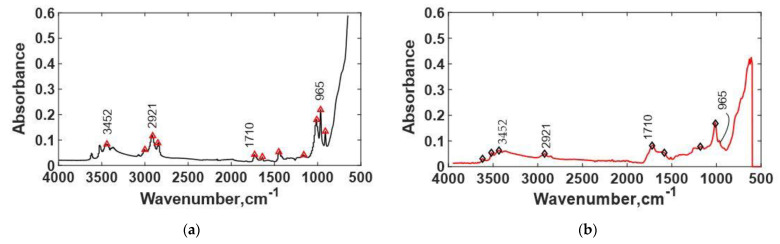
(**a**) FTIR spectra of TIM B preaged, and (**b**) FTIR spectra of TIM B thermal-aged at 125 °C.

FTIR analysis was performed for preaged TIM B and thermal-aged TIM B. The FTIR spectra of TIM B preaged and TIM B thermal-aged at 125 °C are presented in Figure 17. From the FTIR data, it was observed that there is no peak at 1258 cm^−1^, so it indicates that it is silicone-free TIM. It was found that CH_3_ and CH_2_ bonds increase with thermal aging at the 2921 cm^−1^ wavelength, which indicates polymer chain crosslinking. The FTIR also showed the presence of -OH and C=O functional groups at the wavenumbers 3452 and 1710 cm^−1^, respectively. The C=O stretching vibration generally occurred at the 1711 cm^−1^ wavelength [38]. There was a good possibility that the sample went through thermal oxidation during thermal aging, and during thermal aging, C=O bonds degraded, and more hydrogen bonds formed. At peak 965 cm^−1^, for preaged material, there was presence of a carbon–carbon double bond. With the thermal aging, we found that the density of the carbon–carbon double bond decreases. The decrease in the carbon–carbon double bond indicates that more crosslinking occurred. From FTIR results, it was confirmed that the TIM A is a silicone-based material, however, TIM B is a silicone-free elastomer.

### 3.5. Dielectric Properties

**Figure 18:**
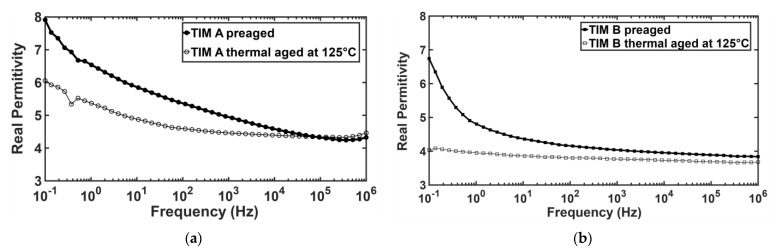
Real permittivity of TM A and TIM B.

The dielectric properties of the thermal interface materials (TIMs) have been measured by broadband dielectric spectroscopy (BbDS). Using this characterization technique, the complex permittivity value has been measured, which has a real and an imaginary part. The real part expresses the capability of the materials to polarize in response to an applied field [39]. The high dielectric constant value indicates the materials to be highly polarized and the low dielectric constant indicates the materials to be insulating. It has been found that the real permittivity values for both TIMs decreased with thermal aging (Figure 18). This can be explained from the view of chemical change of the TIMs due to aging. From FTIR data, it has been observed that polymer chain breaks and oxidation occur during thermal aging. The wavenumber signals at 1000 to 1088 cm^−1^ occur due to asymmetric vibrations of (Si-O-Si), and the corresponding peaks at 950 cm^−1^ are assigned to asymmetric vibrations of (Si-OH) [40]. Due to thermal aging, the Si-O-Si bond density reduced, which may lower the polarizability. The signals at 3435 to 3456 cm^−1^ are attributed to the starching modes of adsorbed water molecules.

**Figure 19:**
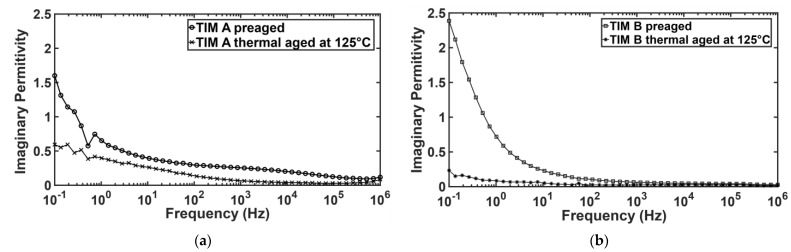
Imaginary permittivity of TIM A and TIM B.

**Figure 20:**
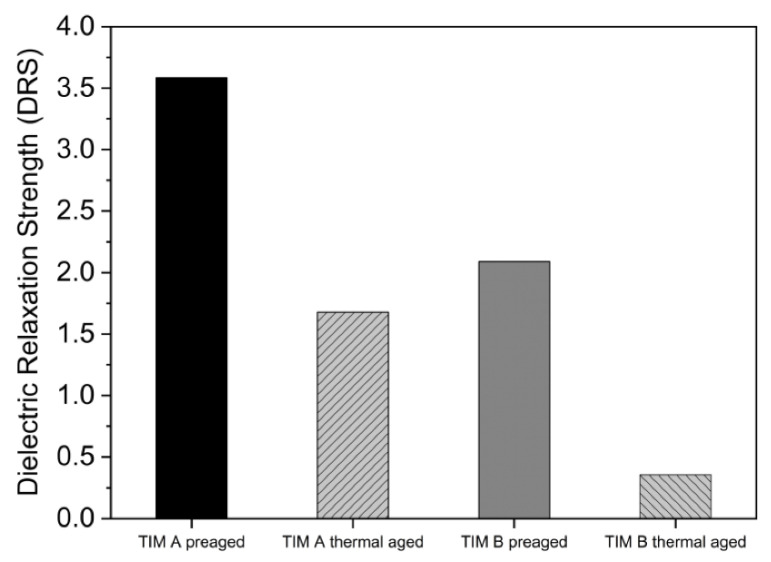
Dielectric relaxation strength (DRS) of TIM A and TIM B.

From FTIR data, it was found that -OH bond density decreased with aging; therefore, the ion movement also decreased, which in turn increased the resistivity of the material. Moreover, the imaginary permittivity also decreased for both TIM A and TIM B samples with thermal aging (Figure 19). The imaginary part indicates the dielectric loss of the material. As the polarization decreases in the sample, the dielectric loss should also be decreased [39]. The dielectric properties, such as the dielectric relaxation strength (DRS), were calculated from real permittivity values for both the TIMs. Dielectric relaxation strength (DRS) is the difference between real permittivity at higher frequency and real permittivity at lower frequency (static permittivity) [26]. DRS values of TIM A and TIM B are shown in Figure 20.

From Figure 20, it can be observed that for both types of samples, the DRS value decreased with thermal aging, which indicates that electrical conductivity decreases with the thermal aging and less polarization occurs inside the material. For the thermally aged sample of TIM B, polymer crosslinking degraded the dielectric property. The polymer chain is not easily polarized due to crosslinking or bulky groups in the chain. On the other hand, for the thermally aged sample of TIM A, the material swells and takes on moisture [41]. Therefore, the thermally aged sample of TIM A shows a relatively higher DRS value than the thermally aged sample of TIM B.

From the overall analysis, the dynamic modulus of thermally aged TIM A increased compared to the preaged TIM A, but remained viscoelastic, which corresponds to the decrease in thermal expansion of the materials and the reduction in the dielectric relaxation strength (DRS). Furthermore, after thermal aging, TIM B samples became hard. Thermal expansion and dielectric relaxation strength (DRS) of thermally aged TIM B were lower than those of preaged TIM B, which corresponds to the increase of dynamic modulus. Finally, the objective of this study was to investigate the change of thermomechanical and dielectric properties of silicone-based and silicone-free TIMs with thermal aging. In addition, the characterization techniques used in this study helped to explain the reasons behind those changes.

## 4. Conclusions

In this work, the impact of thermal aging on the properties of silicone-based and silicone-free thermal gap filler materials has been analyzed, and the effect of the chemical compounds on the change in mechanical, thermal, and dielectric properties has been investigated. After thermal aging, the dynamic modulus of silicone-based thermal gap filler materials increased, but remained viscoelastic, while on the other hand, with thermal aging, the silicone-free thermal gap filler material became hard. In dynamic mechanical analysis, with the increase of temperature, the dynamic modulus of preaged silicone-free thermal gap filler material increased rapidly after 115 °C. It was observed that TIM B becomes hard after thermal aging, which will have an impact on the performance and reliability of the device. In electronic devices, after long-term exposure to heat, silicone-free TIM will become hard, which can create cracks on the adjacent surfaces of the device, which will affect the performance and reliability of the device. Additionally, voids can form as the silicone-free TIM starts to become hard, which will have an impact on the thermal performance of the device. Thermal expansion is higher in silicone-based thermal gap fillers and it increases with temperature, which corresponds to the dynamic modulus results for both preaged and thermal-aged silicone-based thermal gap filler materials. Due to higher thermal expansion, there might be pump-out issues for silicone-based thermal gap filler materials. This phenomenon needs to be considered when designing an electronic package. It is evident from the study that the silicone-based thermal gap filler material has shown better thermal and mechanical properties at higher temperatures than the silicone-free thermal gap filler material. On the other hand, for the applications where electrical insulation is the primary concern, low-k dielectric material such as silicone-free thermal gap filler materials are a better choice of thermal interface material to enhance the performance and reliability of those devices. For silicone-sensitive devices, silicone-free thermal interface materials should be used. Using dielectric relaxation strength (DRS) value, TIMs with required electrical conductivity can be chosen for electronic packages. Depending on the application, the proper thermal interface material needs to be chosen. The results found in this this study can be readily used to design more efficient and reliable electronic packages. Numerical analysis can be performed using the results to find the performance and reliability of the electronic devices designed with these TIMs. Methodologies used in this study can be followed to characterize the mechanical, thermal, and dielectric properties of different kinds of thermal interface materials.

## Data Availability

Not applicable.
